# Histomorphological and inflammatory changes of white adipose tissue in gastrointestinal cancer patients with and without cachexia

**DOI:** 10.1002/jcsm.12893

**Published:** 2021-12-22

**Authors:** Alessio Molfino, Raffaella Carletti, Giovanni Imbimbo, Maria Ida Amabile, Roberta Belli, Cira R.T. di Gioia, Elena Belloni, Francesco Spinelli, Veronica Rizzo, Carlo Catalano, Giuseppe Nigri, Maurizio Muscaritoli

**Affiliations:** ^1^ Department of Translational and Precision Medicine Sapienza University of Rome Rome Italy; ^2^ Department of Surgical Sciences Sapienza University of Rome Rome Italy; ^3^ Department of Radiological, Oncological and Pathological Sciences Sapienza University of Rome Rome Italy; ^4^ Department of Medical‐Surgical Sciences and Translational Medicine Sapienza University of Rome Rome Italy

**Keywords:** Cachexia, Cancer, Adipose tissue, Fibrosis, Inflammation, Body composition

## Abstract

**Background:**

During cancer cachexia, several alterations occur in peripheral tissues, and the adipose tissue may be involved during the catabolic state. We aimed at investigating histological rearrangement and infiltration of inflammatory cells in subcutaneous adipose tissue (SAT) of patients with cancer undergoing surgery, according to the presence/absence of cachexia.

**Methods:**

We considered gastrointestinal cancer patients and controls with non‐malignant diseases undergoing surgery. We collected SAT samples and performed histomorphological analyses [cross‐sectional area (CSA) and per cent of fibrosis] and immunohistochemistry to characterize the inflammatory cells. By computed tomography (CT) scan, we calculated SAT and visceral adipose tissue (VAT).

**Results:**

We enrolled 51 participants (31 gastrointestinal cancer patients and 20 controls). In cancer patients, cachexia was present in 13/31 (42%). The CSA (μm^2^) of the adipocytes from SAT was reduced in cancer patients vs. controls (3148, inter‐quartile range 2574–3755 vs. 4474, inter‐quartile range 3654–5183) (*P* < 0.001), in particular in cachectic patients vs. non‐cachectic (median 2518 vs. median 3470) (*P* = 0.03) and in cachectic vs. controls (*P* < 0.001), as well as in non‐cachectic vs. controls (*P* = 0.04). The median per cent of fibrosis was higher in cancer patients vs. controls (9 vs. 3) (*P* = 0.0001), in particular in cachectic vs. non‐cachectic (13.35 vs. 7.13) (*P* = 0.03). We observed a higher number of macrophages (CD68) (*P* = 0.0001) and T lymphocytes (CD3) (*P* = 0.002) in SAT of cancer patients vs. controls, and the number of T lymphocytes was higher in cachectic vs. non‐cachectic patients (*P* = 0.025). Anorexic cancer patients showed in SAT a higher number of macrophages and T lymphocytes with respect to controls (*P* < 0.0001), whereas no difference was present between anorexic and non‐anorexic patients. At CT scan, cachectic patients showed lower VAT and SAT vs. non‐cachectic (VAT: 97.64 ± 40.79 vs. 212.53 ± 79.24, *P* = 0.0002; SAT: 126.27 ± 87.92 vs. 206.27 ± 61.93, *P* = 0.01, respectively). Cancer patients with low CSA, high degree of fibrosis, and high number of T lymphocytes presented with lower body mass index and lower SAT and VAT at CT scan (*P* ≤ 0.01).

**Conclusions:**

We found histological alterations of SAT among gastrointestinal cancer patients and in particular significant changes in CSA, fibrosis, and inflammation when cachexia was present; the changes in histomorphological parameters of the adipocytes reflected alterations in adiposity at body composition analysis.

## Introduction

Cancer cachexia is considered a multifactorial syndrome mainly characterized by body weight loss associated with wasting of adipose and muscle tissues^1^; it accounts for 50–70% in patients with gastrointestinal cancer and represents a negative prognostic factor in terms of survival, anticancer treatment response, toxicity, and quality of life.^2^

In cancer, tissue wasting and in turn body weight loss are driven by several factors, including anorexia, inflammation, and tumour‐secreted molecules, all determining increased energy expenditure and resulting in a catabolic state.^3^, ^4^, ^5^

The loss of adipose tissue may be considered crucial for the development of cachexia and has been shown to occur early during cancer journey, often anticipating the loss of muscle mass.^6^ In particular, different changes in metabolism and histomorphological alterations of the adipose tissue characterize the wasting condition.^7^ This phenomenon includes the browning, that is, a process of the emergence of beige adipocytes in white adipose tissue, increased lipolysis, and adipokine secretion.^8^ Interestingly, recent data showed that adipose tissue wasting may induce muscle loss, highlighting the relevance of the changes in adipose tissue metabolism in cancer cachexia.^2^, ^6^ In this light, the study of the adipose tissue alterations in patients with cancer may provide important information on the pathophysiology of the metabolic and nutritional derangements observed in this setting. Furthermore, the histomorphological changes, including the inflammatory infiltration, of the adipose tissue represent important features to be considered during cancer cachexia in order to define the altered adipose tissue phenotype.

For this reason, we primarily aimed at assessing the potential differences in histological patterns and in the inflammatory infiltration of the adipose tissue of patients with gastrointestinal cancer with and without cachexia. Secondarily, we evaluated according to the changes of histomorphology and inflammatory infiltration of subcutaneous adipose tissue (SAT) samples the differences in adiposity of cancer patients by body composition analysis.

## Methods

### Study design

This was an observational, case–control study conducted at the Department of Medical‐Surgical Sciences and Translational Medicine and at the Department of Translational and Precision Medicine on a cohort of patients with a new diagnosis of gastrointestinal cancer and on a control group undergoing surgery for malignant and non‐malignant diseases, respectively. The study was performed according to the Declaration of Helsinki and approved by the local ethics committee. Written informed consent was obtained by all the participants of this study. We considered patients with colorectal, gastric, and pancreatic cancer with a recent diagnosis of cancer (≤4 weeks) naïve to any anticancer treatment, eligible for surgical tumour resection. Inclusion criteria included age ≥18 years and the ability to provide informed consent. Exclusion criteria included the presence of a concomitant chronic or acute disease associated with malnutrition (e.g. chronic heart failure, liver cirrhosis, chronic kidney diseases, and infections), cognitive impairment, dysphagia, or occlusion of the gastrointestinal tract; in particular, we have excluded those patients presenting with symptoms of gastrointestinal occlusion due to the possible negative effect on food intake and appetite.

### Participant's nutritional and clinical characteristics

We recorded in all the participants at study visit the current weight and, by patient's self‐report, usual weight and involuntary body weight loss in the prior 6 months, and we calculated the body mass index (BMI). During the study visit, in a fasting state, we collected blood samples in EDTA tubes and then were centrifuged to analyse serum biomarkers such as albumin, total proteins, and C‐reactive protein, and haemoglobin levels with standard automated techniques. We also collected information on the staging and histology of the cancer and on patient's medical history, including co‐morbidities.

### Diagnosis of cachexia

Cancer patients were classified as cachectic (or non‐cachectic) based on the international criteria by Fearon *et al*.^1^ In particular, cachexia was diagnosed when unvoluntary body weight loss over the prior 6 months was >5% (in the absence of simple starvation) or BMI < 20 kg/m^2^ and any degree of weight loss >2%.^1^

We also investigated the presence or absence of anorexia using the validated FAACT questionnaire, which was endorsed by the European Society for Clinical Nutrition and Metabolism.^9^ The cut‐off value we used to diagnose anorexia was ≤30, as previously described.^10^, ^11^

### Histological and morphometric evaluation of subcutaneous adipose tissue

The specimens of SAT (∼1 cm^3^) were collected during the abdominal surgical procedure. In detail, in all the participants, the biopsies were obtained from the SAT located anteriorly to the anterior sheath of the rectus abdominal muscles.

The sample taken was in part immediately frozen in liquid nitrogen and in part included in OCT inside a cryomold and then frozen in liquid nitrogen. Samples were stored at −80°C. Histological sections of frozen adipose tissue from each specimen were performed and stained with haematoxylin–eosin to evaluate morphological changes and the adipocyte cross‐sectional area (CSA). The collagen‐specific Sirius Red staining was used for morphometric analysis of interstitial fibrosis deposition.

All slides with haematoxylin–eosin and Sirius Red‐stained adipose sections (5 μm) were captured with Aperio scanner (Leica Biosystems, Buccinasco, MI, Italy), and then 20 randomly selected images (×200 magnification) were analysed to evaluate adipocyte CSA and interstitial collagen volume fraction with a computerized imaging software (ImageJ, NIH, Bethesda, MD, USA). Four thousand adipocyte cells were counted to evaluate the CSA, and the value was expressed as μm^2^ mean.

The interstitial collagen deposition was automatically calculated as the ratio between red‐stained interstitial area and the total area of the adipose tissue sections and expressed as percentage.

The Sirius Red‐stained sections were also analysed using a light microscope under polarized light at ×400 magnification in 20 randomly selected microscopic fields to evaluate the different types of interstitial collagen content in the adipose tissue. By polarized light microscope, type I collagen appears yellow/orange, while type III collagen appears green.^12^

### Immunohistochemical evaluation of inflammatory infiltration in adipose tissue

The presence of inflammatory infiltrate was evaluated with immunohistochemical stains performed on frozen adipose tissue sections (5 μm). Endogenous peroxidase activity was blocked by 3% hydrogen peroxide. The sections were incubated at room temperature for 1 h, respectively, with mouse anti‐macrophage anti‐CD68 (1:300, mouse monoclonal antibody, clone KP1, ab955, Abcam, Cambridge, UK), mouse anti‐B‐lymphocyte anti‐CD20 (1:100, mouse monoclonal antibody, clone L26, ab9475, Abcam), and rabbit anti‐T‐lymphocyte anti‐CD3 monoclonal antibody (1:100, rabbit monoclonal antibody, clone SP7, ab16669, Abcam), reacting with human samples. Universal Quick Kit, Peroxidase, R.T.U. Staining System (Vector Laboratories, Burlingame, CA, USA) was used to label the primary antibody. The reaction product was visualized with 3,3′‐diaminobenzidine (Vector Laboratories) and counterstaining with Mayer's haematoxylin. Negative control was obtained by omitting the primary antibody. All immunostained slides of adipose tissue sections were captured with Aperio scanner (Leica Biosystems). For each immunostaining, two independent pathologists blinded to the treatment counted the positive cells in 20 randomly selected non‐overlapping fields per section at ×200 magnification and evaluated the mean number of positive cells per field.

### Body composition analysis

Using standard procedure, abdominal fat including total adipose tissue and visceral adipose tissue (VAT) were determined at the level of the third lumbar vertebra (L3) by computed tomography (CT) scan performed with patient in supine position, on a 64‐slice CT scanner (SOMATOM Sensation 64, Siemens Healthineers, Erlangen, Germany), as previously described.^13^ Specifically, quantitative CT measurements were made from existing total‐body CT‐scan image data by qualified radiologists, performed for disease diagnosis and staging. The abdominal fat composition was analysed using a dedicated software (OsiriX Lite, v11.0.3, Bernex, Switzerland). Adipose tissue was quantified semi‐automatically with thresholds between −190 and −30 HU. The SAT area at the same level was calculated by subtracting VAT from total adipose tissue.

### Statistical analysis

We described patients' characteristics using mean ± standard deviation and median with 25th and 75th percentiles for continuous normally and non‐normally distributed variables, as appropriate. Normal distribution was evaluated by Shapiro–Wilk test. Categorical variables were shown as number (%). We evaluated differences among cachectic, non‐cachectic, and controls by analysis of variance and by the Kruskal–Wallis test, as appropriate.

We also used the two‐tailed *t*‐test or Mann–Whitney, according to normal or non‐normal distribution, to evaluate differences between groups. To evaluate potential changes in body composition (adiposity) according to histomorphological parameters, we decided to divide cancer patients in two groups according to the sex‐specific median value of the histological parameters (e.g. low/high CSA) allowing to obtain a balanced distribution between women and men; this approach was performed also because no benchmark of these variables was available in our specific population and because of the reduced sample size. A *P*‐value <0.05 was considered statistically significant.

## Results

### Patient's characteristics

We enrolled 31 gastrointestinal cancer patients (16 men, 52%), 16 with colorectal, 7 with gastric, and 8 with pancreatic cancer with a mean age of 71 ± 12 years undergoing surgery for the neoplastic disease, and 20 controls (8 men, 40%) with a mean age of 58 ± 15 years (*Table* 1), undergoing surgery for benign non‐inflammatory diseases (cholecystectomy for gallstones in 9 patients, abdominal wall surgery for hernia in 8 patients, and 3 patients underwent surgery for cysts—in one patient was ovarian, in one was mesenteric, and in one was subcutaneous cyst). None of the controls was taking anti‐inflammatory medication. Cancer patients were older than controls (*P* = 0.0008). The most common co‐morbidities among the two groups were represented by hypertension, diabetes, and dyslipidaemia (*Table* 1). All cancer patients were naïve to any anticancer treatment at enrolment visit, and none of them were on anti‐inflammatory medication. All the patients did not present with chronic diarrhoea or clear signs of malabsorption.

**Table 1:** Participant's characteristics

Clinical parameter	Gastrointestinal cancer patients (*N* = 31)		Controls (*N* = 20)
	Cachectic (*N* = 13)	Non‐cachectic (*N* = 18)	
Age (years)	66 ± 14	75 ± 9^§^	58 ± 15
Male, *n* (%)	8 (62)	8 (44)	8 (40)
Body weight loss (%)	8.2 (7.1–8.9)	3.2 (1.1–4.1)^#^	0
Anorexia (yes), *n*	10	14	0
BMI (kg/m^2^)	23.9 ± 3.6	27.5 ± 3.3^#^	27.4 ± 4.4
Haemoglobin (g/dL)	12.20 (10.67–12.9)	11.49 (8.95–14)	13.8 (11.8–15.8)
C‐reactive protein (mg/dL)	1.19 (0.17–4.54)	2.91 (0.39–4.04)	0.27 (0.18–0.5)
Albumin (g/dL)	3.35 (2.7–4)	3.4 (3.05–3.58)	4 (3.55–4)
Type of cancer			
Pancreatic, *n* (%)	4 (30)	4 (22)	/
Colorectal, *n* (%)	7 (54)	9 (50)	/
Gastric, *n* (%)	2 (15)	5 (38)	/
Stage I–II, *n*	6	12	
Stage III–IV, *n*	7	6	
Co‐morbidities			
Hypertension, *n* (%)	4 (33)	13 (73)	12 (60)
Diabetes, *n* (%)	0 (0)	7 (39)	6 (33)
Dyslipidaemia, *n* (%)	1 (9)	5 (28)	9 (47)
VAT (cm^2^)	97.64 ± 40.79	212.53 ± 79.24^#^	/
SAT (cm^2^)	126.27 ± 87.92	206.27 ± 61.93^#^	/

BMI, body mass index; SAT, subcutaneous adipose tissue at computed tomography scan; SD, standard deviation; VAT, visceral adipose tissue at computed tomography scan.

Variables are shown as mean ± SD and as median (inter‐quartile range) for non‐normally distributed values.

^§^ *P* = 0.036, cachectic vs. non‐cachectic.

^#^ *P ≤* 0.01, cachectic vs. non‐cachectic.

### Nutritional evaluation

In all cancer patients, we recorded body weight loss in the prior 6 months (median) of 4.14% [inter‐quartile range (IQR) 2.49–7.41]; no changes in body weight were recorded in control group.

Cachexia was diagnosed in 13/31 (42%). In particular, cachexia was present in 7/16 (44%) of colorectal, 4/8 (50%) of pancreatic, and 2/7 (29%) of gastric cancer patients (*Table* 1). No association was detected between the presence of cachexia and the stage of cancer disease. Albumin and C‐reactive protein (CRP) serum levels did not differ between cachectic and non‐cachectic patients (*Table* 1).

No differences in terms of body weight loss (%) and BMI at enrolment visit were present among the different types of cancer (*P* = 0.710 and *P* = 0.843, respectively).

In addition, anorexia assessed by FAACT score was documented in 24/31 (77%) cancer patients. In particular, anorexia was present in 10/16 (63%) of colorectal, 8/8 (100%) of pancreatic, and 6/7 (86%) of gastric cancer patients (*Table* 1).

### Histological and histomorphometric analysis of subcutaneous adipose tissue of cancer patients with and without cachexia and of controls

**Figure 1:**
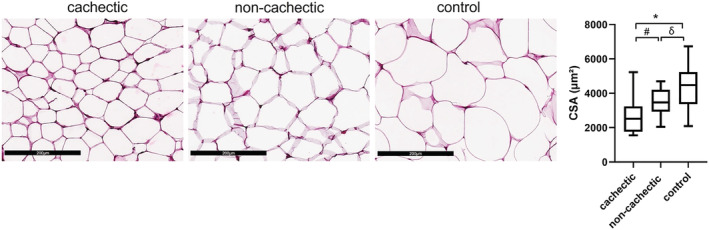
Histomorphometric evaluation of subcutaneous adipose tissue (haematoxylin–eosin ×200). The cross‐sectional area (CSA) of the adipocytes was significantly decreased in cachectic cancer patients compared with non‐cachectic (^#^ *P* = 0.031). CSA was reduced in both cachectic and non‐cachectic patients vs. controls (*P = 0.0001; ^δ^ *P* = 0.040).

The CSA of the adipocytes was significantly reduced in cancer patients with respect to controls (median 3148 μm^2^, IQR 2574–3755 vs. median 4474 μm^2^, IQR 3654–5183) (*P* < 0.001). In particular, the CSA of cancer patients with cachexia (median 2518, IQR 1790–3189) was significantly decreased compared with non‐cachectic cancer patients (median 3470, IQR 2945–4134) (*P* = 0.031) and to controls (*P* = 0.0001), as well as in non‐cachectic vs. controls (*P* = 0.040) (*Figure* 1). No differences were observed among the different types of cancer.

**Figure 2:**
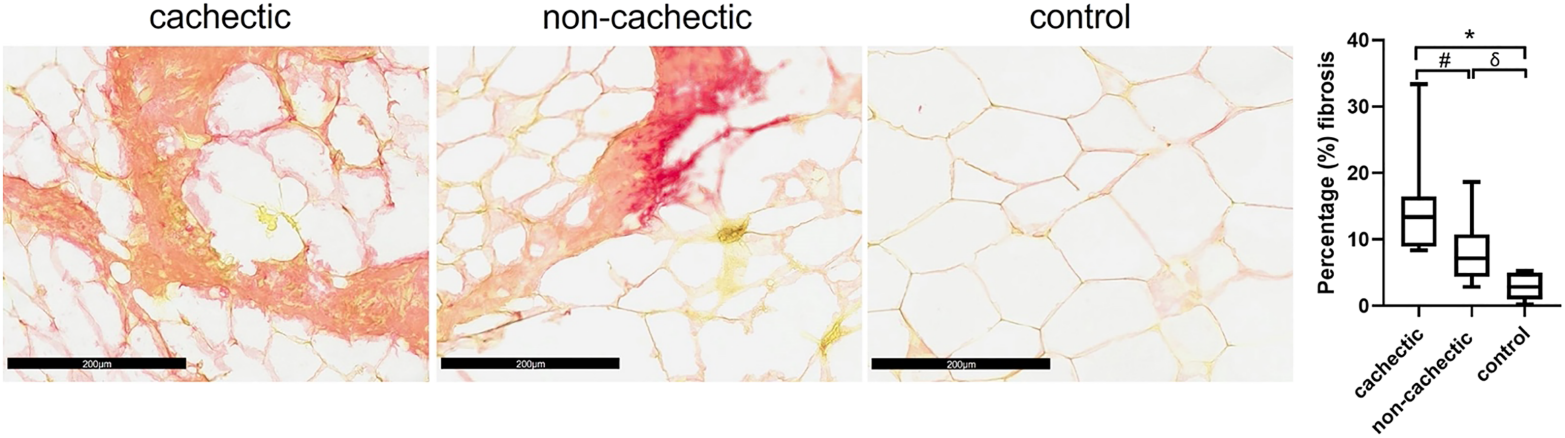
Representative photomicrographs of interstitial fibrosis in subcutaneous adipose tissue (Sirius Red staining ×200). Cachectic cancer patients showed higher interstitial fibrosis vs. non‐cachectic patients (^#^ *P* = 0.033). Higher interstitial fibrosis was present in both cachectic and non‐cachectic patients compared with controls (*P = 0.0001; ^δ^ *P* = 0.001).

Cancer patients presented with a higher degree of fibrosis (%) (median 9.0, IQR 6.63–13.66) with respect to controls (median 3, IQR 0.99–4.99) (*P* = 0.0001). In particular, the per cent of fibrosis deposition was significantly increased in subcutaneous adipose tissue of cachectic cancer patients (median 13.35, IQR 9.29–16.36) compared with non‐cachectic (median 7.13, 4.70–10.01) (*P* = 0.033) and controls (*P* = 0.0001), as well as in non‐cachectic vs. controls (*P* = 0.001) (*Figure* 2).

Both type I and III collagen fibres were represented in increased interstitial fibrous tissue of all cancer patients.

### Inflammatory infiltration in the subcutaneous adipose tissue of cancer patients with and without cachexia and in controls

**Figure 3:**
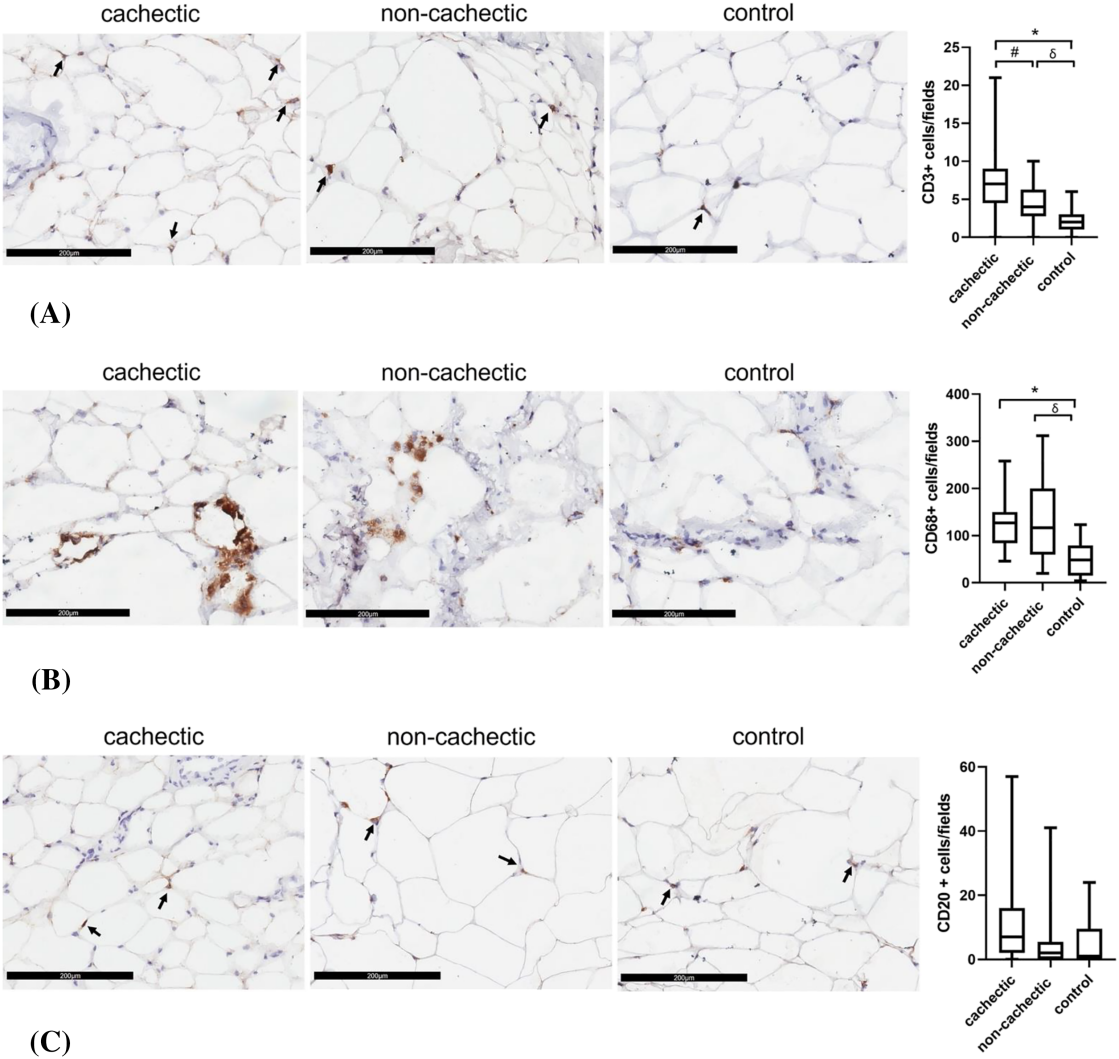
Evaluation of the inflammatory cell infiltration in subcutaneous adipose tissue of cachectic and non‐cachectic cancer patients and controls. (A) The immunostaining photomicrographs (×200) show a significant increase of T‐lymphocyte (CD3^+^) infiltration (arrows) in cachectic cancer patients compared with non‐cachectic (^#^ *P* = 0.025). Both cachectic and non‐cachectic patients showed higher T‐lymphocyte infiltration compared with controls (*P = 0.0001; ^δ^ *P* = 0.005). (B) The immunostaining photomicrographs (×200) showed higher number of macrophage (CD68^+^) infiltration in cachectic and non‐cachectic cancer patients compared with controls (*P < 0.0001; ^δ^ *P* < 0.001). (C) The immunostaining photomicrographs (×200) showed no difference in B‐lymphocyte (CD20^+^) infiltration (arrows) in both cachectic and non‐cachectic patients compared with controls.

In all cancer patients, we found a significant increase in macrophage (CD68^+^, *n*) (128 ± 72) and lymphocyte (CD3^+^, *n*) (6 ± 4) infiltration of the SAT with respect to controls (52 ± 36 and 2 ± 2, respectively) (*P* < 0.0001), without significant difference in the number of B lymphocytes (CD20^+^) (median 4, IQR 1–8 vs. median 1, IQR 1–9) between the two groups. The T‐lymphocyte (CD3^+^) infiltration (median number of cells) was significantly higher in cachectic vs. non‐cachectic patients (7, IQR 5–9 vs. 4, IQR 3–6) (*P* = 0.025) and in cachectic vs. controls (2, IQR 1–3) (*P* = 0.0001), as well as in non‐cachectic compared with controls (*P* = 0.005) (*Figure* 3A). The macrophage (CD68^+^) population showed a significant increase in cachectic patients (126 ± 55) vs. controls and in non‐cachectic (129 ± 83) vs. controls (*P* < 0.0001 and *P* < 0.001, respectively), whereas no differences were found between cachectic and non‐cachectic patients (*P* = 0.458) (*Figure* 3B). The macrophages showed several cytoplasmatic lipid droplets and a characteristic crown‐like disposition around adipocytes. No differences were observed in the B‐lymphocyte (CD20^+^) infiltration between cachectic, non‐cachectic, and controls (*Figure* 3C). No differences were found in the inflammatory infiltration of SAT according to the type of cancer and sex. In cancer patients, no significant correlation was found between per cent of body weight loss and the total number of lymphocytes (*r* = 0.295, *P* = 0.107).

### Anorexia and inflammatory infiltration in the subcutaneous adipose tissue of cancer patients with and without cachexia and controls

Based on the presence/absence of anorexia, we observed a higher number of macrophages and T lymphocytes in subcutaneous adipose tissue of anorexic cancer patients (131.79 ± 77.27 and 6.25 ± 4.72, respectively) with respect to controls (*P* < 0.0001) and a higher number of macrophages in non‐anorexic (114.71 ± 50.31) vs. controls (*P* = 0.001), whereas no differences were found in T lymphocytes between non‐anorexic (4 ± 2.38) and controls (*P* = 0.130). The number of macrophages and T lymphocytes did not differ between anorexic and non‐anorexic patients (*P* > 0.20).

In cancer patients, we also observed a negative correlation between FAACT score and the total number of lymphocytes (*r* = −0.419, *P* = 0.021).

### Body composition analysis and histomorphological changes of subcutaneous adipose tissue among cancer patients

At CT‐scan analysis, cancer patients showed VAT and SAT of 163.9 ± 86.8 and 172.42 ± 82.85, respectively. Cachectic patients showed lower VAT (*P* = 0.0002) and SAT (*P* = 0.01) with respect to non‐cachectic patients (*Table* 1).

Cancer patients with low CSA of the adipocytes (below the sex‐specific median value) presented with lower SAT (*P* = 0.012), VAT (*P* = 0.002), and BMI (*P* = 0.009) with respect to patients with high CSA (over sex‐specific median value) (*Table* 2). Cancer patients with high degree of fibrosis (over the sex‐specific median value) of SAT presented with lower SAT and VAT and BMI with respect to cancer patients with low degree of fibrosis (below the sex‐specific median value) (*P* ≤ 0.01) (*Table* 2). Patients with higher number of T lymphocytes (CD3^+^) (over the sex‐specific median value) in SAT samples presented with decreased SAT (*P* = 0.01) and VAT (*P* = 0.01), as well as decreased BMI (*P* = 0.0019) compared with cancer patients with lower number of T lymphocytes (below the sex‐specific median value).

**Table 2:** Differences among cancer patients in SAT and VAT (by CT scan) and in BMI, according to histomorphological parameters and inflammatory patterns at adipocyte biopsy

	Low CSA	High CSA	*P*	Low fibrosis	High fibrosis	*P*	Low CD3^+^	High CD3^+^	*P*	Low CD20^+^	High CD20^+^	*P*	Low CD68^+^	High CD68^+^	*P*
SAT (cm^2^)	133 ± 80	212 ± 68	**0.01**	217 ± 56	128 ± 83	**0.004**	212 ± 51	133 ± 91	**0.0128**	183 ± 56	160 ± 108	0.48	175 ± 78	169 ± 92	0.86
VAT (cm^2^)	113 (56–139)	203 (149–242)	**0.002**	188 (144–229)	113 (56–140)	**0.008**	203 (144–242)	117 (93–140)	**0.01**	172 ± 78	155 ± 99	0.62	144 ± 114	188 ± 117	0.203
BMI (kg/m^2^)	24 ± 4	28 ± 3	**0.0096**	28 ± 3	24 ± 4	**0.0097**	28.0 ± 2.9	23.9 ± 3.5	**0.0019**	26.8 ± 3.5	25.3 ± 4.0	0.301	24.9 ± 22.7	27.4 ± 24.3	0.079

BMI, body mass index; CSA, cross‐sectional area; CT, computed tomography; SAT, subcutaneous adipose tissue; VAT, visceral adipose tissue.

Bold indicates statistically significant *P*‐values.

No difference was observed in body composition parameters according to number of B lymphocytes (CD20^+^) and macrophages (CD68^+^) of SAT samples (*Table* 2).

## Discussion

The data obtained by our study indicate that poor nutritional status is highly prevalent among patients with gastrointestinal cancer undergoing surgery. In particular, although in our cohort the median body weight loss in the previous 6 months was below 5%, the diagnosis of cachexia, based on the international criteria,^1^ was performed in more than 40% of the patients. As expected, based on recent literature,^14^ the highest prevalence of cachexia was detected in patients with pancreatic cancer, although this specific subgroup did not show greater body weight loss or lower BMI compared with colorectal or gastric cancer patients. Also, anorexia that represents an important component of the catabolic status of gastrointestinal cancer patients^10^, ^14^ was highly prevalent in our cancer group, particularly among pancreatic cancer patients.

As for the primary aim of our study, we characterized the adipose tissues changes in terms of histology and inflammatory infiltration, and we documented that the CSA of the adipocytes obtained by SAT biopsy during surgery was reduced in patients with gastrointestinal cancer compared with controls suggesting a clinically relevant wasting condition in this setting. This histological aspect was confirmed in cachectic patients in whom CSA was significantly reduced with respect to non‐cachectic. These data are in line with those obtained by Batista *et al*.,^7^ who documented in SAT of gastrointestinal cancer patients with cachexia a decrease of adipocyte size, measured as sectional area and cell perimeter, with respect to individuals undergoing surgery for non‐neoplastic disease.^7^

In cancer cachexia, adipose tissue atrophy was previously described both in animal models and in humans.^15^, ^16^ The wasting process of the adipocytes in cancer cachexia may be determined by several factors including enhanced lipolysis,^15^ and browning phenomenon,^2^ actively contributing to promote systemic and local (adipose tissue) catabolic state.

In addition, our data indicate that a higher degree of fibrosis was present in cancer patients when compared with controls. Regarding this, we acknowledge the potential confounding factor of age, considering that cancer patients were older than controls. However, the fibrosis deposition was significantly higher in patients with cachexia compared with those without cachexia.

Fibrosis is an indicator of an uncontrolled remodelling of extracellular matrix, and this may be a key component of the altered adipose tissue metabolism of cancer patients.^17^ Moreover, Alves *et al*. documented that fibrosis of adipose tissue was associated with a derangement of transforming growth factor‐β pathway showing an up‐regulation of transforming growth factor‐β and SMAD protein expression in SAT of patients with cancer cachexia.^16^

Interestingly, our results confirm the high grade of inflammatory infiltration of SAT in gastrointestinal cancer patients. In particular, cachectic patients showed a higher number of T lymphocytes with respect to non‐cachectic patients and to controls. This observation was confirmed in terms of number of macrophages resulting higher in patients with cachexia versus controls, whereas no differences were seen according to the presence/absence of cachexia. Importantly, adipose tissue represents a relevant source of inflammatory mediators, which may play a role in promoting cancer cachexia. In this light, our data may suggest a role of T lymphocytes in promoting cachexia more than B lymphocytes and macrophages. Interestingly, recent data suggest a protective role of macrophages in the loss of adipose tissue during cancer.^13^ However, the role of inflammatory cells of the adipose tissue during cancer is not yet fully clarified, and the different immune cells at the different proportions between cachectic, non‐cachectic, and controls may reveal important pathophysiological implications of inflammation in determining changes (reduction) in adiposity in cancer cachexia.

Inflammation is a potent determinant of metabolic and nutritional derangements in cancer, and it may induce changes peripherally and systemically acting also at central nervous system level, leading to anorexia.^11^, ^18^, ^19^ In this light, we analysed the inflammatory infiltration of the adipose tissue, stratifying our patients also based on the presence/absence of anorexia, not observing differences between anorexic and non‐anorexic patients in terms of macrophages and T‐lymphocyte infiltration likely due to the small number of individuals in each of the two groups. However, experimental data indicate that during cancer cachexia, activation of central nervous system pathway (GFRAL–RET) determines the expression of genes involved in adipose tissue wasting.^20^ Also, anorexic patients did not show differences in terms of CRP levels compared with non‐anorexic, and, although apparently unexpected, this is not unusual considering that anorexia may be the result of a neuroinflammation, which is not reflected by the circulating levels of inflammatory markers.^11^ In fact, hypothalamic pro‐inflammatory cytokines, among other factors, are key in triggering the development of anorexia.^4^, ^18^, ^21^

Moreover, by our study, we were able to collect novel information regarding the changes in adiposity (VAT and SAT assessed by CT scan) and their relation with the histomorphological changes of SAT in cancer patients. This aspect appears crucial for physicians considering that adipose tissue changes may occur in the early stage of cachexia even when body weight loss and inflammatory status (e.g. increased CRP levels) is minimal. In particular, we documented that patients with low CSA and high degree of fibrosis of the SAT showed lower SAT and VAT when compared with patients with high CSA and low degree of fibrosis. Also, patients with higher T‐lymphocyte infiltration in the adipocytes samples showed lower VAT and SAT at body composition analysis. We believe that our data are novel and provide relevant information on the correlation analysis linking the SAT and VAT values with adipose tissue alterations (i.e. CSA, per cent of fibrosis, and inflammatory infiltration).

In particular, Han *et al*.^22^ previously assessed the impact of VAT and SAT changes in cachectic gastric cancer patients in terms of prognosis, revealing that indexes of SAT can be used as an independent prognostic factor (overall survival) among patients with gastric cancer with cachexia,^22^ although no information was available in terms of histomorphological changes. However, in recently diagnosed cancer patients with cachexia, other authors observed a selective decrease in visceral white adipose tissue.^23^ In addition, in a retrospective analysis, it was shown that a rapid decline in VAT over 30 days was related with poor outcomes (reduced survival) among patients affected by an unresectable pancreatic cancer.^24^ This information highlights the clinical relevance of a short‐term assessment of body composition analysis (changes in adiposity) in patients with gastrointestinal cancer that should guide physicians for an early nutritional intervention.

In comparison with prior data obtained among cancer patients with cachexia in terms of adipocytes changes,^7^ our study added important clinical information represented mainly by the differences in terms of VAT and SAT at CT scan.

In particular, we believe that it is interesting that cachectic patients in our cohort presented differences in terms of VAT and SAT since the time of first cancer diagnosis (as for our inclusion criteria) and that our results documenting wasting processes of the adipocytes in a single biopsy may reflect the changes observed in adipose tissue depots.

We acknowledge the limitations of our study. In particular, the changes observed in the adipocytes of cachectic patients were obtained by a selective population of cancer patients (gastrointestinal) with potential important clinical differences between gastric vs. colorectal vs. pancreatic cancer patients. Also, we did not find difference in terms of CRP or albumin concentration among cachectic and non‐cachectic patients and controls, and this could be at least in part an effect of the small sample size and in part likely due to the high percentage of patients with an early stage of cancer disease; in fact, we enrolled patients at their first cancer diagnosis (new diagnosis) and that were eligible for surgery and did not receive any anticancer treatments. The changes in histomorphology and inflammation observed in SAT samples may not be representative of other adipose tissue compartments, such as VAT. However, we have also analysed these results according to the changes in body composition, including VAT, assessed by CT scan. By our results, we did not report histomorphological and inflammatory changes of the adipocytes in patients with high body weight loss (i.e. greater ≥10%). Regarding the inflammatory profile of SAT, we did not perform flow cytometry in order to confirm the phenotype of immune cells, and further studies should include this analysis.

Finally, although our study design was cross‐sectional, we believe that a longitudinal analysis may inform on how these changes impact on strong outcomes, including survival.

In conclusion, by our study, we documented several histological alterations of SAT among gastrointestinal cancer patients and specifically changes in CSA, per cent of fibrosis, and in terms of inflammatory patterns when cachexia was diagnosed.

Moreover, we may assert that the changes in histomorphological parameters of the adipocytes reflect alterations in adiposity at body composition analysis that represent a clinically relevant problem in cancer patients to be early diagnosed and treated to improve cancer patient's outcome and quality of life.

## Funding

A.M. received for this study a research grant provided by the Sapienza University of Rome (Sapienza Università di Roma) (Grant Number RG11816427B021CF). R.C. and R.B. contributed to this study as recipient of the PhD programme in Innovative Biomedical Technologies in Clinical Medicine, Sapienza University of Rome.

## Conflict of interest

None declared.
